# The EQ-5D and EQ-HWB fit the perceptions of quality of life from a Chinese perspective: a concept mapping study

**DOI:** 10.1186/s12955-025-02361-3

**Published:** 2025-03-31

**Authors:** Yifan Ding, Zhuxin Mao, Zhihao Yang, Siliu Feng, Jan Busschbach

**Affiliations:** 1 Department of Psychiatry, Erasmus MC, University Medical Center Rotterdam, Rotterdam, the Netherlands; 2 Centre for Health Economics Research and Modelling Infectious Diseases, Vaccine and Infectious Disease Institute, University of Antwerp, Antwerp, Belgium; 3 Health Services Management Department, Guizhou Medical University, Guiyang, China

**Keywords:** Quality of life, EQ-5D, EQ-HWB, Cultural impact, Concept mapping

## Abstract

**Background:**

The escalating globalization of health assessments underscores a pivotal challenge: Do Quality of Life (QoL) instruments, developed primarily in Western contexts, accurately reflect the perspectives of non-Western populations? This study evaluates the alignment of EQ-5D and EQ-HWB items with QoL dimensions identified in Chinese literature, and compares QoL perceptions between Chinese residents and those living abroad who may be influenced by Western values.

**Methods:**

This study employed three successive rounds of participant recruitment to refine the concept mapping process. Initially, 13 bilingual QoL experts in the Netherlands piloted the methodology, providing feedback on item translation and expression. Subsequently, 18 Chinese expatriates in the Netherlands, with partial education abroad, reviewed the revised materials to represent perspectives influenced by Western culture. Finally, 20 native Chinese residents, who were born and educated in China, formed the target group. Utilizing feedback from the pilot stage, a pool of 54 QoL items derived from Chinese literature, plus an additional eight from the EQ-HWB, were assembled. The Group Concept Mapping (GCM) method was used, with participants organizing the items to reflect their interrelationships. Data were analyzed via Groupwisdom™, an online tool supporting multidimensional scaling (MDS) and cluster analysis, culminating in visual cluster rating maps that highlighted the item associations and groupings.

**Results:**

In China, a five-cluster MDS map was identified: personal abilities, family and society, physical health, mental health, and self-cognition (stress value: 0.183), with physical health prioritized. Abroad, four clusters appeared: mental health, social connections, daily activities, and physical function (stress value: 0.185), prioritizing social connections. The EQ-5D missed the "family and society" cluster in China and "social connections" abroad. In contrast, the EQ-HWB covered all clusters in both groups.

**Conclusions:**

The EQ-5D items align well with the somatic and partially with the mental clusters, while the EQ-HWB also covers the adaptation cluster and the remaining mental cluster aspects. Both instruments reflect the QoL perceptions of Chinese individuals, although EQ-5D focuses more on health than overall well-being. Cultural differences affect priorities: participants in China value physical health most, whereas those abroad emphasize social connections.

**Supplementary Information:**

The online version contains supplementary material available at 10.1186/s12955-025-02361-3.

## Introduction

The ongoing debate about the cultural appropriateness of Quality of Life (QoL) instruments in non-Western settings highlights critical concerns regarding their universal applicability. Most QoL tools currently in use, including those employed in China, originate from Western scientific and cultural contexts[1–3]. However, it is commonly postulated that cultural differences influence how QoL is conceptualized, casting doubt on the content validity of Western-developed instruments in non-Western contexts [2, 4].

The tension between standardization and cultural relevance is exemplified by the EQ-5D and EQ-HWB instruments. While the EQ-5D is the most widely used health-related QoL (HRQoL) measure globally, its narrow focus on physical and mental health domains has prompted calls for adaptations, such as a China-specific ‘bolt-on’ version [5–7]. Meanwhile, the newer EQ-HWB broadens its scope to include social care and well-being [8]. However, both tools originate from Western contexts, which has sparked discussions about the necessity for culturally specific modifications [4, 8]. On the other hand, excessive adaptation poses challenges for the cross-cultural interpretation of results: if different instruments are used in different cultures, compatibility and comparability of the results are compromised. Acknowledging these cultural disparities, it becomes evident that the use of standardized tools is crucial. Standardization ensures valid and reliable QoL measurements and maintains uniformity across studies, regions, and populations [9]. This uniformity is essential for informing evidence-based policy decisions, planning healthcare services, and ensuring equitable resource allocation. Moreover, it provides vital health indicators necessary for effective public health interventions [10].

In response to these challenges, we recently did a systematic review identified HRQoL items (distinct from general QoL) prioritized by the Chinese population [11]. Building on these results, the presents study tests whether these items related sufficiently with the items from the EQ-5D and EQ-HWB. While the former study we focused on HRQoL, in the present we take a broader perspective on QoL as include both items of the healthcare oriented EQ-5D and the EQ-HWB which designed to assess QoL across both healthcare and social care sectors [8]. Using Group Concept Mapping (GCM), the present study classifies QoL dimensions derived from Chinese literature and evaluates their alignment with the items from both the EQ-5D and EQ-HWB. Our objectives are to: 1) Assess how well the EQ-5D and EQ-HWB items correspond with QoL items prioritized in Chinese contexts; and 2) Whether culturally tailored adaptations of these instruments are warranted.

## Methods

### Study design and pilot study

Between 2023 and 2024, we conducted a GCM study with Chinese participants in both the Netherlands and China. The study aimed to categorize HRQoL items identified from an extensive literature review within a Chinese cultural context. Additionally, we incorporated items from EQ-5D and EQ-HWB to ensure a comprehensive evaluation of all relevant QoL aspects.The items were initially translated from English to Chinese by YD and subsequently reviewed by ZM. Following this, a pilot study was conducted involving bilingual professionals proficient in both English and Chinese. These professionals helped refine the whole data collection process and provided suggestions for improving the translations. After an internal discussion of all the suggestions, we finalized the materials for formal data collection including all the items extracted from the systematic review, EQ-5D and EQ-HWB. The study received ethical approval from the Health Services Management Department, Guizhou Medical University (2024–40).

### Participants

Informed consent was obtained from all participants to ensure ethical compliance and awareness. Three distinct participant groups were recruited to refine the concept mapping process: 1) Pilot Group: This group consisted of Chinese public health professionals with experience studying or working in the Netherlands. They tested the study procedures, including clustering items and performing the GCM task, to ensure clarity of instructions and identify potential issues. As bilingual participants, they reviewed both English and Chinese translations of the items, providing feedback to refine the materials. 2) A group Chinese individuals born in China and currently pursuing higher education in the Netherlands were asked to participate in testing the adjustments based on the input of the pilot group. This purposively sampled group was relatively young, highly educated, and non-specialists in QoL, representing individuals influenced by Western culture. Their participation further tested the study's feasibility, with data collection proceeding smoothly. 3) This group consisted of general Chinese individuals born and educated in China, representing the broader target population for this investigation. Recruited through snowball sampling, this group included participants with diverse educational backgrounds, offering a wider perspective on QoL perceptions.

### Group concept mapping

GCM is a widely used method for exploring complex phenomena and generating new insights. Based on Trochim's methodology, it involves four main steps: (1) preparation and item generation, (2) structuring and rating items, (3) data analysis, and (4) data interpretation [12]. While there is no strict upper limit on participants, a minimum of 10 participants is recommended to ensure robust and meaningful results [12].

#### Step 1: preparation and generating items

The researchers began by selecting participants and defining the focus of the study. Instead of conducting a traditional brainstorming session to generate new statements, QoL-related items were derived from a previously published systematic review. The translations of these items were validated by bilingual team members (YD, ZM, and ZY).

We initially identified 60 items from the literature review. Following the pilot test, we refined this list by eliminating duplicate items and merging similar ones based on the recommendations from the Pilot study group. This refinement process led us to finalize a set of 54 items for clustering. The original items and the selection process are detailed in Appendix 1.

The initial plan was to incorporate 5 items from the EQ-5D and 25 from the long version of the EQ-HWB into the clustering process. We began by cross-referencing these items with the 54 QoL items derived from Chinese literature, finding considerable overlap. Ultimately, only eight unique EQ-HWB items that captured novel dimensions not present in the literature-based pool were added. These items are 'feel unsafe', 'frustrated', 'had nothing to look forward to', 'have no control over day-to-day life', 'feel unable to cope with day-to-day life', 'feel good about yourself', 'do the things you wanted to do', and 'feel accepted by others', increasing the total to 62 items. Each item is detailed in Appendix 2, annotated to indicate whether it originates from EQ-5D or EQ-HWB.

#### Step 2: structuring and rating items

Typically, the structuring and rating processes would be managed via an online program. However, due to the program's inability to support simplified Chinese, we resorted to face-to-face data collection. Participants physically sorted printed cards into piles based on similarities, categorizing them under the theme of ‘health’. The rules for sorting are: 1) an item can only go into one group; 2) there must be multiple groups, and each group should contain more than one item.

Additionally, participants labeled each group according to their own understanding and subsequently rated the items on a 5-point Likert scale, ranging from "very unimportant" to "very important," based on perceived importance to their health.

#### Step 3: data analysis and interpretation

The data were combined and analyzed using the GroupWisdom tool, an online GCM program. Results were visualized through cluster rating maps, which utilized two-dimensional non-metric multidimensional scaling (MDS) and cluster analysis of a similarity matrix [13, 14]. Each point on the map represented a statement, positioned based on how participants grouped it with others [15]. Statements closer together were more frequently sorted together, while those farther apart were less frequently associated [16].

Cluster maps were generated based on the proximity of points, with each cluster representing a distinct theme [17]. During iterative clustering, statements were combined into fewer clusters containing more items. At each iteration, researchers (YD, JB) evaluated the thematic coherence of the clusters, and the final number of clusters was determined by consensus. Cluster names were derived from the participants’ labels, with researchers selecting the most appropriate label for each cluster. If any clusters were not represented by EQ-5D or EQ-HWB items, this indicated potential gaps in these instruments. Missing clusters that were important or misaligned with the questionnaire’s goals raised concerns about content validity.

Model fit was assessed using the stress value, which ranges from 0 to 1, with lower values indicating a better fit of the map to the similarity matrix [18]. The acceptable range for concept map stress values is between 0.13 and 0.36 [19].

## Results

### Participants

We conducted the formal analysis with two arms: one in China and one in the Netherlands. Demographic details are provided in Table 1. In China, we recruited 20 lay participants from seven provinces. One participant's understanding of the sorting task was unclear, so only her rating task result was included. In the Netherlands, we recruited 18 participants who complete both sorting and rating tasks. The sample from the Netherlands had a higher level of education and was younger overall.

**Table 1:** Socio-demographic characteristics of the sample

Variable	Group	Samples of participants	
		In China (*N* = 20)	In the Netherlands (*N* = 18)
Sex	Female	14	11
	Male	6	7
Highest level of Education	High school	1	
	Technical college	8	
	Bachelor and above	11	18
Age	20–30	12	10
	31–40	2	8
	41–50	3	
	51–60	2	
	> 60	1	

### Results from participants in China

Based on the sorting results from the Chinese participants, we selected a MDS map displaying five clusters. Additional clusters did not reveal any meaningful contextual differences. These five clusters were named using suggestions from the Group Wisdom program, informed by participants' input. After thorough discussion, the authors reached a consensus on naming the five principal QoL clusters as follows: personal abilities, family and society, physical health, mental health, and self-cognition. The self-cognition cluster, which includes elements such as concentration, a sharp mind, fatigue, and pain, was identified as the most challenging to interpret. The final MDS map recorded a stress value of 0.183. This suggests not only high data quality and reliability but also that the clusters or groupings of health concepts were logically coherent, underscoring a consistent understanding of 'health' among laypeople, which aligns well with the adjusted terminology used in health-related decision-making.

**Fig. 1:**
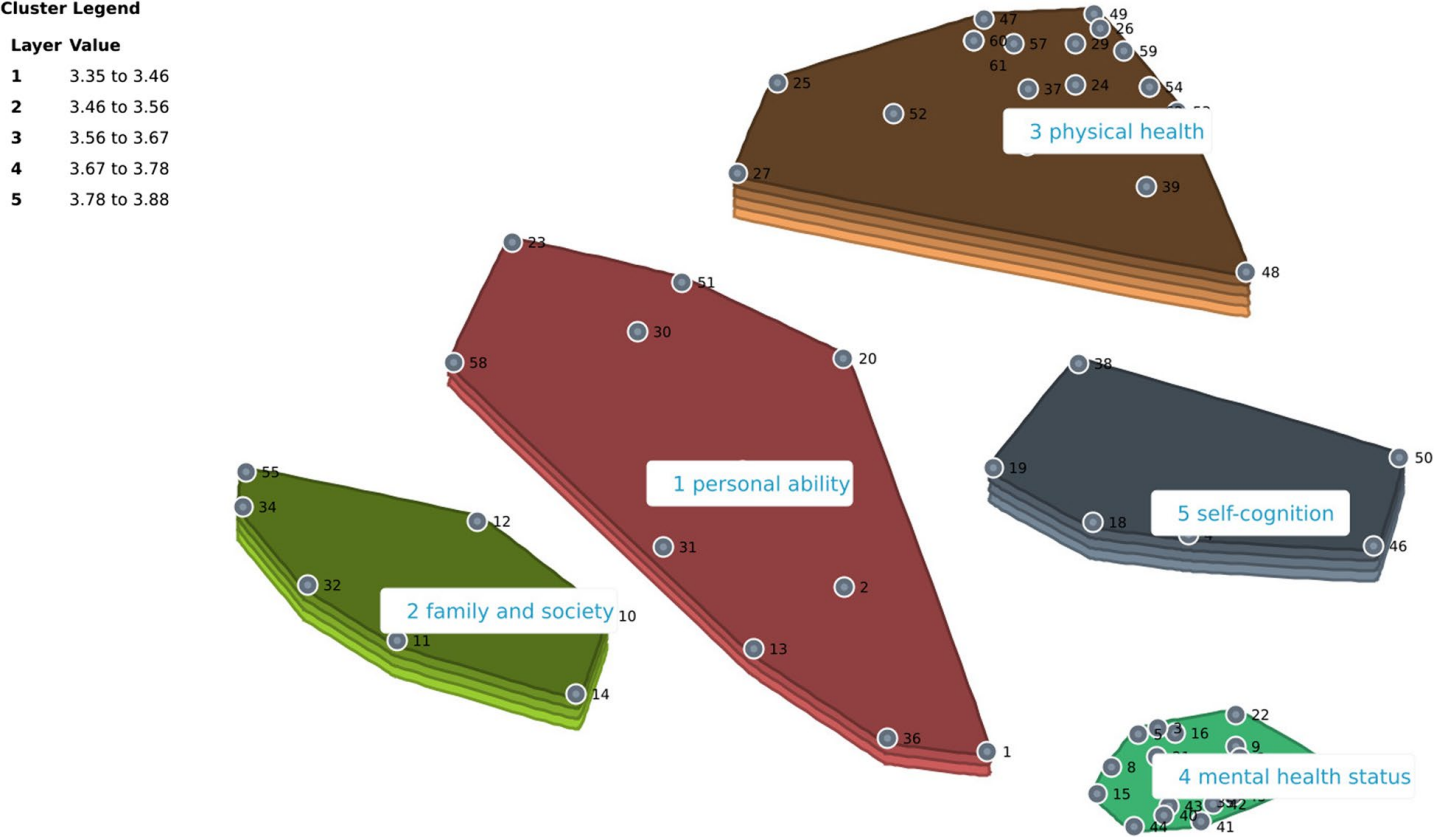
Cluster rating map of participants in China

Figure 1 in the results section illustrates the cluster rating map for China, showing both sorting and rating outcomes. The depth of layers in each cluster indicates the perceived importance of the items within—more layers signify greater importance [18]. Based on these findings, Cluster 3 (physical health), Cluster 2 (family and society), and Cluster 5 (self-cognition) were deemed most important. Conversely, Cluster 4 (mental health) was rated as the least important.

Table 2 provides the average bridging values for each cluster, with lower values indicating items that were frequently sorted together, thus suggesting stronger thematic coherence [19]. Here, Cluster 4 (mental health) exhibited the highest coherence, with a very low bridging value of 0.066, indicating strong internal consistency. Conversely, Cluster 2 (family and society) showed the least coherence. Detailed information on the items, including their average importance ratings and bridging values, is presented in Appendix 3.

**Table 2:** Overview of cluster information of people in China

No	Cluster	Number of items	Mean bridging value	Bridging value range
1	personal ability	11	0.506	0.263–0.87
2	family and society	8	0.795	0.549–1
3	physical health	19	0.217	0.042–0.483
4	mental health status	18	0.066	0–0.323
5	self-cognition	6	0.399	0.279–0.617

### Results from participants in Netherlands

From the sorting results with participants in the Netherlands, we derived a MDS map featuring four distinct clusters: mental, society connections, daily activities, and physical function. The final MDS map recorded a stress value of 0.185, indicating a good fit with the model.

**Fig. 2:**
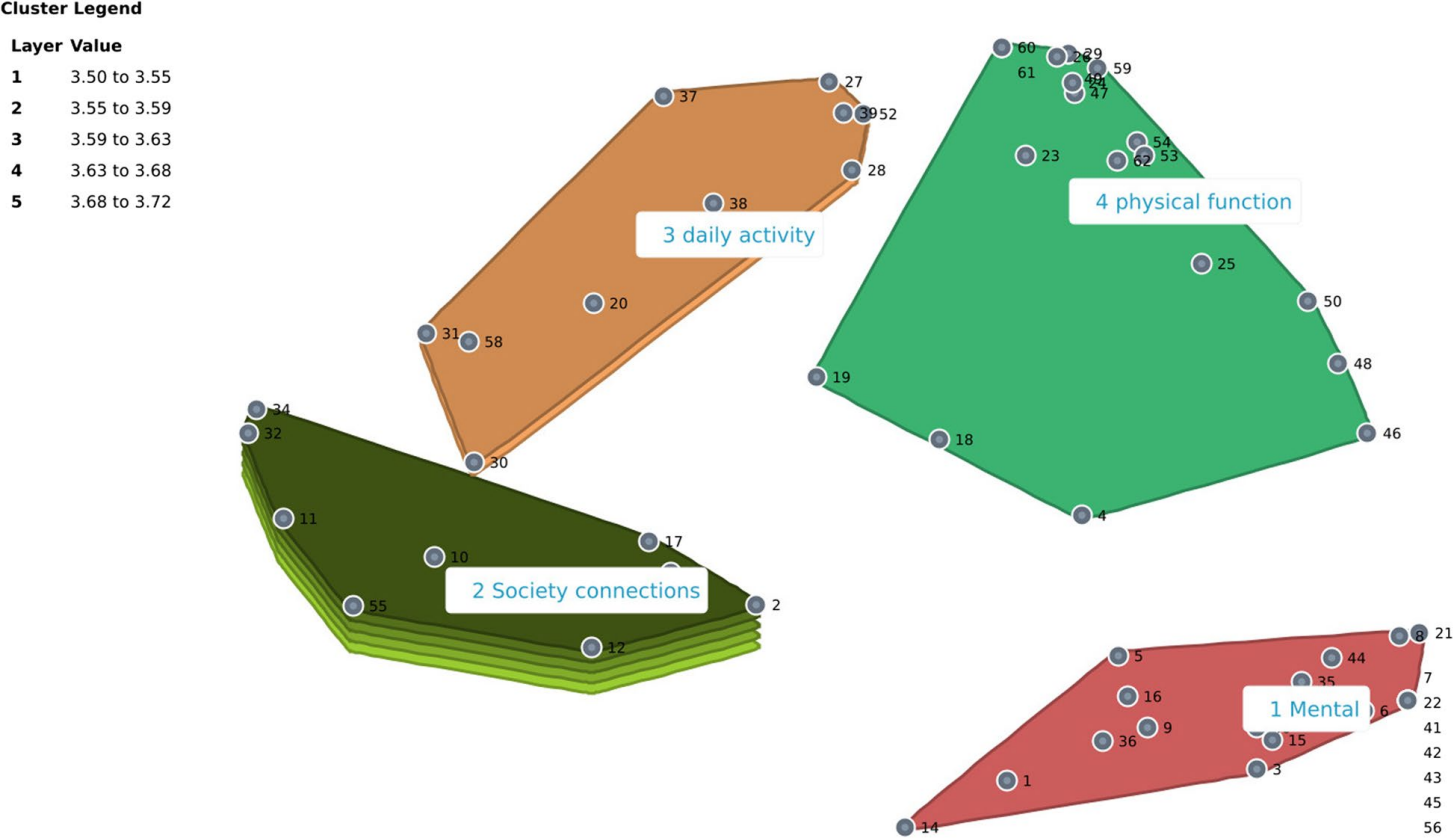
Cluster rating map of participants in Netherlands

Figure 2 displays the cluster rating map, showing the relative importance assigned to each cluster. Cluster 2 (society connections) was rated as the most important. In contrast, Clusters 1 (mental) and 4 (physical function) were perceived as less important.

Table 3 details the average bridging values for each cluster, which measure how often items within the same cluster were grouped together, reflecting thematic coherence. Cluster 1 (mental) exhibited the highest thematic coherence, with the lowest bridging value of 0.218, indicating strong internal consistency within this cluster. Conversely, Cluster 2 (society connections) showed the lowest coherence. Further details on the specific items, including their average importance ratings and bridging values, are provided in Appendix 4.

**Table 3:** Overview of cluster information of people in Netherlands

No	Cluster	Number of Items	Mean bridging value	Bridging value range
1	mental	21	0.218	0–0.802
2	society connections	10	0.839	0.684–1
3	daily activity	11	0.612	0.378–0.855
4	physical function	20	0.377	0.095–0.701

### EQ-5D and EQ-HWB item classification and importance in both groups

Table 4 presents the classification of items from the EQ-5D and the EQ-HWB (long version) as categorized by participants from both China and the Netherlands. In this table, items from the EQ-5D are highlighted in a gray box, while items from the EQ-HWB are in a white box. This layout allows for clear visualization of how each item aligns with the identified clusters in each group.

**Table 4:** Distribution of EQ-5D and EQ-HWB items in sorting results and importance

Items	Importance	Participants in Netherlands	Items	Importance	Participants in China
mobility	4.78	daily activity	feel unsupported	3.2	family and society
self-care	4.67	daily activity	anxiety	4.15	mental health status
usual activities	3.78	daily activity	depression	3.75	mental health status
mobility	4.78	daily activity	no control over day-to-day life	3	mental health status
self-care	4.67	daily activity	anxiety	4.15	mental health status
usual activities	3.78	daily activity	frustrated	3.15	mental health status
memory	3.78	daily activity	depression	3.75	mental health status
concentration	3.72	daily activity	nothing to look forward to	3.2	mental health status
anxiety	3.50	mental	loneliness	2.9	mental health status
depression	3.89	mental	feel unsafe	3.25	mental health status
no control over day-to-day life	3.83	mental	feel good about myself	3.45	mental health status
anxiety	3.50	mental	self-care	4.2	personal ability
frustrated	2.89	mental	usual activities	3.75	personal ability
depression	3.89	mental	self-care	4.2	personal ability
had nothing to look forward to	3.67	mental	usual activities	3.75	personal ability
loneliness	3.44	mental	feel accepted by others	2.75	personal ability
feel unsafe	3.22	mental	do the things I wanted to do	3.65	personal ability
feel accepted by others	3.22	mental	mobility	4.15	physical fitness
feel good about myself	4.17	mental	discomfort	4.3	physical fitness
pain	4.06	physical function	vision	4.05	physical fitness
discomfort	3.94	physical function	hearing	4.45	physical fitness
vision	3.89	physical function	mobility	4.15	physical fitness
hearing	3.22	physical function	memory	3.9	physical fitness
unable to cope with day-to-day life	4.22	physical function	sleep	4.65	physical fitness
clear mind	3.94	physical function	discomfort	4.3	physical fitness
sleep	4.50	physical function	pain	3.55	self-cognition
exhausted	3.17	physical function	unable to cope with day-to-day life	3.9	self-cognition
pain	4.06	physical function	concentration	3.5	self-cognition
discomfort	3.94	physical function	clear mind	4.05	self-cognition
feel unsupported	3.67	society connections	exhausted	3.85	self-cognition
do the things I wanted to do	3.89	society connections	pain	3.55	self-cognition
Mean of all items	3.56		Mean of all items	3.65	
Mean of items of EQ-5D	4.09		Mean of items of EQ-5D	3.98	
Mean of items of EQ-HWB	3.79		Mean of items of EQ-HWB	3.70	

Analysis reveals that the EQ-5D items cover four out of the five clusters identified by participants from China and three out of the four clusters identified in the Netherlands. On the other hand, items from the EQ-HWB are represented across all clusters in both groups. It is noteworthy that the clusters not covered by the EQ-5D specifically relate to social interactions—namely, ‘family and society’ in the China sample and ‘society connections’ in the Netherlands sample.

Furthermore, the average importance scores for the EQ-5D items are above the overall average scores for all items among participants from both arms. However, the importance scores for the EQ-HWB items are only marginally higher. This suggests that while the EQ-5D effectively captures many aspects of QoL deemed important by the participants, the EQ-HWB provides a more comprehensive representation, especially in the context of social clusters.

## Discussion

We identified four and five interpretable clusters of QoL, varying based on the sample—either Chinese civilians residing in China or abroad. All clusters were represented by items from the EQ-HWB, while the EQ-5D omitted one cluster in both samples. Notably, the clusters missed by the EQ-5D emphasize social interactions: ‘family and society’ in the China sample and ‘social connections’ in the Netherlands sample. This discrepancy is consistent with the fundamental purposes of the two instruments: the EQ-5D primarily focuses on direct health outcomes, whereas the EQ-HWB is designed to encompass broader aspects of well-being, including social care. Additionally, the average importance scores for all EQ-5D items were higher than the average scores for all items among participants from both regions, although the EQ-HWB’s scores were only slightly higher. These findings contribute to the ongoing debate regarding whether the EQ-5D and EQ-HWB align with lay perspectives on QoL in China. This investigation underscores that while the EQ-5D and EQ-HWB effectively cover the identified dimensions of QoL, the EQ-5D's focus remains predominantly on health, whereas the EQ-HWB also captures the social dimensions of well-being.

As indicated in the introduction, there is ongoing debate about the ability of the EQ-5D and EQ-HWB to fully capture the general population’s perspective on QoL in China, as their development have relied more heavily on expert judgments and literature reviews in western contexts rather than direct input from Chinese cultural contexts. The positive alignment between the conceptual frameworks of these instruments and the perspectives of Chinese lay participants support the validity of their development methods in China. This suggests that the concept maps created by Chinese participants logically reflect the intended goals of both instruments, reinforcing their relevance across cultural settings. If misalignment had occurred, it could indicate inadequacies in representing QoL within the Chinese context. However, the observed consistency demonstrates that Chinese laypeople can effectively articulate QoL using the provided items. This highlights the adaptability of the EQ-5D and EQ-HWB frameworks in capturing diverse cultural perspectives on health and well-being.

Cultural and regional variations play a substantial role in shaping health policy design. When standardized tools do not sufficiently reflect the diverse health needs of different populations, there is a risk that economic evaluations may not fully capture the nuances of these groups, potentially leading to an exacerbation of health disparities. Consequently, it is advisable to adapt health policies to align with the distinct socio-economic and health profiles of various communities, thereby facilitating more effective and equitable health interventions across a range of cultural settings.

In our study, the Netherlands sample, which comprised solely Chinese international students, rated social connections as the most crucial dimension. This emphasis likely stems from several factors. Firstly, both family and social interactions are fundamental for health [20, 21], as they provide essential social support that contributes to better physical and mental health outcomes [22–24]. Social support also acts as a protective factor against harmful behaviors and emotional distress [25]. The prominence of social dimensions in our findings can be attributed to the specific challenges faced by these students, including depression, stress, anxiety, insomnia, culture shock, loneliness, language barriers, and difficulties adjusting to new social and academic environments [26]. Such challenges predominantly impact social aspects of health, rather than physical, and significantly hinder their cultural integration and academic achievements [26]. The support from family and connections within their own cultural community plays a pivotal role in alleviating these difficulties by offering a familiar social context and practical assistance, which is invaluable for navigating life abroad. Moreover, considering that these students are typically young and physically healthy, physical health issues may be less of a concern compared to their social and mental well-being. This perspective shifts the focus towards enhancing their social support networks as a key area for improving their overall QoL.

Although both the Chinese and Netherlands groups rated the importance of social aspects highly, they regarded mental health dimensions relatively low. In the Chinese sample, mental health was the lowest-rated dimension, while it ranked third out of four in the Netherlands group. This discrepancy might be attributed to several factors: (1) visibility and detection challenges: Mental health issues are often less visible and harder to detect than physical health problems [27]. Mental disorders typically manifest as changes in emotions, thoughts, and behaviors, affecting individuals' relationships with themselves and others, rather than presenting clear physical symptoms [28]. (2) stigma and societal attitudes: In both the Netherlands and more significantly, in China, mental health issues are still heavily stigmatized. Among university students, a population with higher education levels, the stigma around mental health issues is so pronounced that only 18% to 34% of those suffering from severe depression or anxiety seek professional help [29]. This stigma likely discourages individuals from acknowledging or addressing their mental health problems [30]. The observed attitudes toward mental illness and differences could be related to cultural factors [31–33]. Yang et al. [34, 35] found that stigma towards mental illnesses among Chinese people is particularly influenced by cultural norms rooted in Confucianism. A key principle of Confucianism dictates that every individual must adhere to the moral demands of society to maintain personal and social harmony. Consequently, those with mental illnesses, who may struggle to meet these societal expectations, are often viewed with skepticism regarding their moral status [36]. (3) resource scarcity and societal neglect: The lack of mental health resources further complicates this issue. It is estimated that around 130 million adults in China suffer from mental disorders annually, yet the majority do not receive any treatment [37, 38]. With a lifetime prevalence of 16.6% for mental disorders in China, the economic impact is substantial, characterized by high treatment costs and reduced productivity[37, 39]. The shortage of mental health professionals, such as psychiatrists, psychologists, and social workers, exacerbates this lack of care and reinforces the perception that mental health is not a priority [40]. A rather cynical interpretation could suggest that the lower prioritization of mental health in our study aligns with a broader societal neglect. It is possible that individuals rate mental health aspects lower because they believe they have more control over mental health problems than physical health issues. It's important to note that our study did not measure the QoL of individuals with mental health conditions but rather asked healthy people to prioritize aspects of health, where they now deem physical aspects more important than mental.

Defining the 'self-cognition' cluster within the Chinese sample was challenging due to the term's ambiguity. The program-generated label 'self-cognition' included a mix of items that ranged from cognitive functions like 'sharp mind' and 'concentration' to those linked to physical sensations such as 'fatigue' and 'pain,' as well as elements reflecting emotional well-being, notably 'unable to cope.' This blend of cognitive, physical, and emotional aspects made it difficult to assign a precise overarching label. Participant suggestions varied widely, proposing terms from 'work' and 'lifestyle' to broader concepts like 'life' and 'self-emotions.' After thoughtful consideration, we retained 'self-cognition' because it best captured the QoL and well-being dimensions influenced by an individual's sense of self. Although we believe that 'self-awareness'—a synonym identified by the authors with a similar meaning but better clarity—may be easier to understand than 'self-cognition', we chose not to rename the 'self-cognition' cluster ourselves. This decision was made to preserve the participants' original grouping logic and maintain fidelity to their conceptualization of QoL. This cluster, while seemingly ambiguous, emerged naturally from participant sorting patterns. It includes both cognitive (e.g., concentration) and physical (e.g., pain) items, embodying a holistic understanding of self-awareness. This approach is rooted in Chinese cultural contexts, where cognitive and bodily states are viewed as interconnected. This reflection of health also mirrors the principles of Traditional Chinese Medicine (TCM) [11], which espouses a holistic view of health. This holistic view is also reflected in clusters with high bridging values such as 'family and society' in the local Chinese sample and 'social connections' in the Netherlands group. High bridging values suggest a broad conceptual scope. For example, the item such as 'morality' maintains coherence within the 'Society Connection' cluster, although it potentially overlaps with clusters like 'Daily Activity'. This configuration highlights the interconnectedness of QoL dimensions in real-world contexts.

Based on importance ratings, we found that the average importance scores for both the EQ-5D and EQ-HWB were higher than the overall average for all items across both participant groups. This observation suggests that, despite identifying a broader range of health-related concepts in the literature, the most critical items had already been incorporated into the EQ-5D and EQ-HWB. The reason why these instruments did not capture many additional concepts in our study likely stems from their primary focus on specific health outcomes. In contrast, our study adopted a more comprehensive approach, exploring all aspects of health, which led to the identification of a wider array of concepts. Although the EQ-5D covers fewer clusters than the EQ-HWB, this comes at a cost for the EQ-HWB—its average importance scores are lower than those of the EQ-5D. This indicates that while the EQ-HWB provides broader coverage, the EQ-5D has been particularly successful in selecting a smaller number of highly pertinent items, which resonates strongly with the general public’s concerns about health.

Compared to the existing frameworks in China, a systematic review reveals that people perceive QoL from two primary perspectives: TCM and Modern Medicine (MM) [11]. The TCM framework includes five domains—physical health, mental health, natural environment, social environment, and emotions—while the MM framework comprises four domains: physical health, mental health, social health, and environment. Our findings indicate that the classification of QoL in China and the Netherlands generally aligns well with these two frameworks, with one notable exception: items related to the natural environment are classified into different clusters. Specifically, 'climate adaptation and adjustment' are consistently categorized under 'personal ability' and 'daily activity' in both groups, likely because adapting to weather is seen as a demonstration of personal adaptability, a quality required daily. Conversely, 'dwelling conditions' are placed within the 'social connections' cluster, possibly because dwelling conditions are often associated with neighborhood and personal relationships.

Our study has several limitations. First, although the sample sizes of 20 participants in China and 18 in the Netherlands meet the minimum requirements for Group Concept Mapping (GCM), they may still limit the stability and generalizability of the cluster configurations. Reassuring is that our 'stress values'—0.183 for China and 0.185 for the Netherlands—fall within the acceptable range of 0.13 to 0.36 [19], indicating that the clusters are reliably configured. However, employing larger and more diverse samples could further enhance the generalization of our findings. Second, the demographic skew towards younger, highly educated individuals, primarily PhD candidates in the Netherlands, may introduce selection bias, affecting the generalizability to a broader Chinese expatriate population. This demographic might overemphasize social connections due to acculturative stress, as indicated by our findings on unique social stressors faced by expatriates. Third, we adjusted terminology from 'QoL' to 'health' during the pilot phase to aid comprehension, and did not collect participants' health status, limiting further analysis. Additionally, technical limitations of GroupWisdom, including its inability to display Chinese characters, necessitated a shift from an online to a face-to-face data collection format, followed by manual data entry into GroupWisdom.

Despite these challenges, GCM effectively identified culturally relevant QoL clusters.

However, the exploratory nature of this method calls for further validation. Future studies should employ psychometric methods, such as factor analysis or item response theory (IRT), to statistically validate the dimensional structure of these clusters. For instance, testing the internal correlations of items within the 'family and society' and 'social connections' clusters Confirmatory Factor Analysis (CFA) would further explore the structure of these clusters.

## Conclusion

The EQ-HWB successfully captures all clusters of lay people's understanding of health in China, while the EQ-5D misses those specifically related to social interactions. This discrepancy highlights the EQ-5D’s primary focus on health outcomes, contrasting with the EQ-HWB’s broader emphasis on well-being and social care. The high average importance scores for both instruments suggest that their developers effectively captured the key aspects of health and well-being from the perspective of laypeople, aligning well with the general population’s health priorities.

## Supplementary Information

Supplementary Material 1.

Supplementary Material 2.

Supplementary Material 3.

Supplementary Material 4.

## Data Availability

Data is available on reasonable request.

## Abbreviations

QoL: Quality of Life
EQ-HWB: EQ Health and Well-being
HRQoL: Health Related Quality of Life
GCM: Group Concept Mapping
MDS: Multidimensional scaling
TCM: Traditional Chinese Medicine
MM: Modern Medicine
IRT: Item Response Theory
CFA: Confirmatory Factor Analysis

## References

[1] Mao Z, et al. Similarities and differences in health-related quality-of-life concepts between the east and the west: a qualitative analysis of the content of health-related quality-of-life measures. Value Health Reg Issues. 2021;24:96–106. 33524902 10.1016/j.vhri.2020.11.007

[2] Li M, et al. Culture-related health disparities in quality of life: assessment of instrument dimensions among Chinese. Front Public Health. 2021;9: 663904. 34178922 10.3389/fpubh.2021.663904 PMC8221419

[3] Yang F, et al. Do rural residents in China Understand EQ-5D-5L as Intended? Evidence from a qualitative study. Pharmacoecon Open. 2021;5(1):101–9. 32285402 10.1007/s41669-020-00212-z PMC7895880

[4] Mao Z, et al. Exploring subjective constructions of health in China: a Q-methodological investigation. Health Qual Life Outcomes. 2020;18(1):165. 32493342 10.1186/s12955-020-01414-z PMC7268713

[5] Mao Z, et al. Developing and testing culturally relevant bolt-on items for EQ-5D-5L in Chinese populations: a mixed-methods study protocol. BMJ Open. 2024;14(1): e081140. 38286698 10.1136/bmjopen-2023-081140 PMC10826542

[6] Cheuk Wai Ng C, Wai Ling Cheung A, Lai Yi Wong E. Exploring potential EQ-5D bolt-on dimensions with a qualitative approach: an interview study in Hong Kong SAR, China. Health Qual Life Outcomes. 2024;22(1):42. 38816769 10.1186/s12955-024-02259-6 PMC11141055

[7] Liao M, Wu H, Yang Z, Huang Y, Janssen MF, Bonsel G, Luo N. Testing four cognition bolt-on items to the EQ-5D in a general Chinese population. Eur J Health Econ. 2025;26(3):403–11. 10.1007/s10198-024-01714-x 39162893

[8] Zhang G, et al. Can items derived from international literature be used in national quality of life instruments? A qualitative study conceptualising the EQ-HWB in China. J Patient Rep Outcomes. 2024;8(1):83. 39102010 10.1186/s41687-024-00767-z PMC11300404

[9] Reeve BB, et al. ISOQOL recommends minimum standards for patient-reported outcome measures used in patient-centered outcomes and comparative effectiveness research. Qual Life Res. 2013;22(8):1889–905. 23288613 10.1007/s11136-012-0344-y

[10] Diseases GBD, Injuries C. Global burden of 369 diseases and injuries in 204 countries and territories, 1990–2019: a systematic analysis for the Global Burden of Disease Study 2019. Lancet. 2020;396(10258):1204–22. 33069326 10.1016/S0140-6736(20)30925-9 PMC7567026

[11] Ding Y, et al. Differences and common ground in the frameworks of health-related quality of life in traditional Chinese medicine and modern medicine: a systematic review. Qual Life Res. 2024;33(7):1795–806. 38740639 10.1007/s11136-024-03669-1 PMC11176225

[12] Jackson KM, Trochim WMK. Concept mapping as an alternative approach for the analysis of open-ended survey responses. Organ Res Methods. 2002;5(4):307–36.

[13] Hosseini N, et al. Factors affecting clinicians’ adherence to principles of diagnosis documentation: A concept mapping approach for improved decision-making. Health Inf Manag. 2022;51(3):149–58. 33845621 10.1177/1833358321991362

[14] Trochim W, Kane M. Concept mapping: an introduction to structured conceptualization in health care. Int J Qual Health Care. 2005;17(3):187–91. 15872026 10.1093/intqhc/mzi038

[15] Kabukye JK, de Keizer N, Cornet R. Elicitation and prioritization of requirements for electronic health records for oncology in low resource settings: A concept mapping study. Int J Med Inform. 2020;135: 104055. 31877404 10.1016/j.ijmedinf.2019.104055

[16] Klappe ES, et al. Effective and feasible interventions to improve structured EHR data registration and exchange: A concept mapping approach and exploration of practical examples in the Netherlands. Int J Med Inform. 2023;173: 105023. 36893655 10.1016/j.ijmedinf.2023.105023

[17] Trochim WM, McLinden D. Introduction to a special issue on concept mapping. Eval Program Plann. 2017;60:166–75. 27780609 10.1016/j.evalprogplan.2016.10.006

[18] Kane M, Trochim WMK. Concept mapping for planning and evaluation. Thousand Oaks: Sage Publications, Inc; 2007.

[19] Donnelly JP. A systematic review of concept mapping dissertations. Eval Program Plann. 2017;60:186–93. 27693034 10.1016/j.evalprogplan.2016.08.010

[20] Michaelson V, Pilato KA, Davison CM. Family as a health promotion setting: A scoping review of conceptual models of the health-promoting family. PLoS ONE. 2021;16(4): e0249707. 33844692 10.1371/journal.pone.0249707 PMC8041208

[21] Stapley E, et al. A Scoping review of the factors that influence families’ ability or capacity to provide young people with emotional support over the transition to adulthood. Front Psychol. 2021;12: 732899. 34721198 10.3389/fpsyg.2021.732899 PMC8555465

[22] Winetrobe H, et al. Differences in health and social support between homeless men and women entering permanent supportive housing. Womens Health Issues. 2017;27(3):286–93. 28153741 10.1016/j.whi.2016.12.011 PMC5435523

[23] Hwang SW, Chambers C, Katic M. Accuracy of self-reported health care use in a population-based sample of homeless adults. Health Serv Res. 2016;51(1):282–301. 26119335 10.1111/1475-6773.12329 PMC4722213

[24] Shaheen AM, et al. Perceived social support from family and friends and bullying victimization among adolescents. Child Youth Serv Rev. 2019;107: 104503.

[25] Yang YC, et al. Social relationships and physiological determinants of longevity across the human life span. Proc Natl Acad Sci. 2016;113(3):578–83. 26729882 10.1073/pnas.1511085112 PMC4725506

[26] Hussain M, Shen H. A study on academic adaptation of international students in China. High Educ Stud. 2019;9(4):80–91.

[27] Fang Z, et al. Mental health in China: exploring the impacts of built environment, work environment, and subjective perception. Front Psychol. 2024;15: 1352609. 38455120 10.3389/fpsyg.2024.1352609 PMC10918749

[28] Jones PB. Adult mental health disorders and their age at onset. Br J Psychiatry. 2013;202(s54):s5–10. 10.1192/bjp.bp.112.119164 23288502

[29] Gulliver A, Griffiths KM, Christensen H. Perceived barriers and facilitators to mental health help-seeking in young people: a systematic review. BMC Psychiatry. 2010;10: 113. 21192795 10.1186/1471-244X-10-113 PMC3022639

[30] Corrigan P. How stigma interferes with mental health care. Am Psychol. 2004;59(7):614–25. 15491256 10.1037/0003-066X.59.7.614

[31] Haraguchi K, et al. Stigma associated with schizophrenia: cultural comparison of social distance in Japan and China. Psychiatry Clin Neurosci. 2009;63(2):153–60. 19335384 10.1111/j.1440-1819.2009.01922.x

[32] Schomerus G, et al. Public attitudes towards mental patients: a comparison between Novosibirsk, Bratislava and German cities. Eur Psychiatry. 2006;21(7):436–41. 16531016 10.1016/j.eurpsy.2006.01.009

[33] Wong DF, et al. Family burdens, Chinese health beliefs, and the mental health of Chinese caregivers in Hong Kong. Transcult Psychiatry. 2004;41(4):497–513. 15709648 10.1177/1363461504047932

[34] Yang LH, et al. “What matters most:” a cultural mechanism moderating structural vulnerability and moral experience of mental illness stigma. Soc Sci Med. 2014;103:84–93. 24507914 10.1016/j.socscimed.2013.09.009

[35] Yang LH, Kleinman A. “Face” and the embodiment of stigma in China: the cases of schizophrenia and AIDS. Soc Sci Med. 2008;67(3):398–408. 18420325 10.1016/j.socscimed.2008.03.011 PMC2911783

[36] Yin H, et al. Mental health stigma and mental health knowledge in Chinese population: a cross-sectional study. BMC Psychiatry. 2020;20(1):323. 32571270 10.1186/s12888-020-02705-x PMC7310154

[37] Huang Y, et al. Prevalence of mental disorders in China: a cross-sectional epidemiological study. Lancet Psychiatry. 2019;6(3):211–24. 30792114 10.1016/S2215-0366(18)30511-X

[38] Phillips MR, et al. Prevalence, treatment, and associated disability of mental disorders in four provinces in China during 2001–05: an epidemiological survey. Lancet. 2009;373(9680):2041–53. 19524780 10.1016/S0140-6736(09)60660-7

[39] Xu J, et al. The economic burden of mental disorders in China, 2005–2013: implications for health policy. BMC Psychiatry. 2016;16:137. 27169936 10.1186/s12888-016-0839-0 PMC4864926

[40] Xu Z, et al. The state of mental health care in China. Asian J Psychiatr. 2022;69: 102975. 34998231 10.1016/j.ajp.2021.102975

