# Supplementary material for: The EQ-5D and EQ-HWB fit the perceptions of quality of life from a Chinese perspective: a concept mapping study
Source: Health Qual Life Outcomes. 2025 Mar 31;23:29. doi: 10.1186/s12955-025-02361-3 (PMC11959868; doi:10.1186/s12955-025-02361-3)
Supplement: Supplementary file 1 — Supplementary Material 1. [file 12955_2025_2361_MOESM1_ESM.docx]

Appendix 1 original items and selection process

| Original item in English | Translated in Chinese |  |  |
| --- | --- | --- | --- |
| stool | 粪便（例如：次数是否正常；干稀是否正常） |  |  |
| verbal expression | 语言表达能力（例如：是否能清楚表达自己的想法等） |  |  |
| family (e.g.support；fights) | 家庭关系（例如：家庭支持；家庭矛盾等） |  |  |
| urinate | 小便（例如：次数是否正常；颜色是否正常） |  |  |
| consciousness | 头脑清醒 | 合并(combined) | clear mind |
| grief | 悲伤 |  |  |
| constitution | 体质 |  |  |
| spirit of eye | 眼神（例如：目光是否有神；眼神转动是否灵活等） |  |  |
| pensiveness | 沉思 | 合并(combined) | worry |
| sleep | 睡眠质量 |  |  |
| mobility | 行动能力；走动能力（如果平常使用拐杖或轮椅，那么按照使用时的情况来回答） |  |  |
| sound/hearing | 听力 |  |  |
| appetite | 食欲 |  |  |
| self-care | 自理能力，照顾自己的能力（例如，擦洗，穿衣，上厕所等） |  |  |
| discomfort | 感到不舒服，不适（例如：感觉想吐，喘不上气，瘙痒等；但不包括疼痛） |  |  |
| energy | 精力充沛 |  |  |
| pain | 感到疼痛 |  |  |
| complexion/color of face | 气色（例如：面色是否有光泽，口唇润泽等） |  |  |
| fatigue/exhausted | 疲劳 |  |  |
| joy/happy mood | 开心 |  |  |
| anxiety | 焦虑 |  |  |
| (ability) to think | 思考能力 | 合并(combined) | clear mind |
| worry | 担忧；忧虑 |  |  |
| confidence | 信心；自信 |  |  |
| **fear** | 恐惧 |  |  |
| anger | 生气；愤怒 |  |  |
| memory | 记忆力 |  |  |
| concentration | 专注力 |  |  |
| loneliness | 感到孤独 |  |  |
| ability to complete work and study | 完成工作和学习的能力 |  |  |
| sex life | 性生活 |  |  |
| dwelling conditions | 居住条件 |  |  |
| economic conditions/status | 经济条件 |  |  |
| satisfaction | 对自己的生活感到满意 |  |  |
| good relationships and communication | 与他人的关系和交流 | 合并(combined) | social interaction |
| climate adaptation and adjustment | 气候适应能力(例如，对居住地气候的适应性；对季节气候变化的适应性等) |  |  |
| daily activities | 日常活动能力（例如，家务，休闲活动及娱乐，购物，旅行等） |  |  |
| heavy physical work | 重体力活（例如：搬运重物等） |  |  |
| body strength | 体力 |  |  |
| vision | 视力 |  |  |
| body weight | 体重 |  |  |
| family medical history | 家庭用药史 |  |  |
| physical exercise and play | 身体锻炼 | play的部分放在了日常活动中(The 'play' component is incorporated into daily activities.) |  |
| dependence on medication | 药物依赖（例如：日常生活是否需要依靠药物或医疗的帮助） |  |  |
| depression | 抑郁 |  |  |
| stress | 压力 |  |  |
| regularity in daily life | 日常生活的规律性 |  |  |
| morality | 品行；道德 |  |  |
| no worry | 没有压力 | 合并(combined) | worry |
| clear mind | 清晰的头脑或思维（可以清晰地思考） |  |  |
| positive attitude | 积极的态度（例如：积极的心态或生活态度） |  |  |
| no pressure | 没有压力 | 合并(combined) | stress |
| sharp mind | 清晰的头脑或思维（可以快速反应） |  |  |
| peace | 内心平和 |  |  |
| breadth of mind | 心胸宽广（例如：包容，慷慨等） |  |  |
| social support | 社会支持 |  |  |
| feel unsafe | 感到不安全（如：害怕跌倒，身体伤害或受到虐待） |  |  |
| ability to make decisions | 做决定的能力；我能自己做决定 |  |  |
| adaptability to social environment | 社会环境适应性（例如：应对不良刺激，适应嘈杂环境，与人打交道等） |  |  |
| frustrated | 沮丧 |  |  |
| social interactions | 社交（例如：与他人良好的关系或交流） |  |  |
| burdens to others | 是否感到自己是他人的负担 |  |  |
| had nothing to look forward to | 感觉没有什么事情可以期待 |  |  |
| have no control over day-to-day life | 是否能够掌控自己的日常生活（例如：是否可以选择自己做什么，或者别人对你做什么） |  |  |
| feel unable to cope with day-to-day life | 是否能够应对自己的日常生活 |  |  |
| feel good about yourself | 是否对自己感觉不错 |  |  |
| do the things you wanted to do | 是否能够做自己想做的事情 |  |  |
| feel accepted by others | 是否感到被他人接受（例如：是否能够做自己并且感到有归属感） |  |  |
