# Supplementary material for: The EQ-5D and EQ-HWB fit the perceptions of quality of life from a Chinese perspective: a concept mapping study
Source: Health Qual Life Outcomes. 2025 Mar 31;23:29. doi: 10.1186/s12955-025-02361-3 (PMC11959868; doi:10.1186/s12955-025-02361-3)
Supplement: Supplementary file 2 — Supplementary Material 2. [file 12955_2025_2361_MOESM2_ESM.docx]

**提示语:**

**任务一**：在分类任务中受访者会被告知接下来要进行一个分类任务，以下这些卡片都是关于对‘健康’的描述。请大家根据自己对健康的理解，对这些卡片进行分类。具体步骤如下：首先，将自己感觉表达或形容的是一类东西的卡片放在一摞。在分好类后需要给每一摞分好类的卡片起一个能够代表它们表达的主题的名字。在分类过程中按照自己的理解来操作即可，没有对错之分。

**任务二：**在评分任中，需要请大家对每一张卡片上的内容对自己的健康的重要程度进行评分。评分标准如下：1分：最不重要；2分：不太重要；3分：一般重要；4分：比较重要；5分：最重要。请大家根据自己的判断和感受，逐一对每张卡片进行评分。

Instructions in English:

Task 1: In the sorting task, participants will be informed that they are about to carry out a classification task. The following cards all contain descriptions related to 'health.' Please sort these cards based on your own understanding of health. The specific steps are as follows: First, place the cards that you feel describe or represent the same type of thing in one pile. After sorting, you will need to give each pile a name that reflects the theme expressed by the cards in that group. Sort the cards based on your own understanding; there are no right or wrong answers.

Task 2: In the rating task, participants will be asked to rate the importance of the content on each card to their own health. The rating scale is as follows:

1 = Least important

2 = Not very important

3 = Moderately important

4 = Quite important

5 = Most important

Please rate each card one by one according to your own judgment and feelings.

**Concept mapping items translation:**

| **Original item in English** | **Translated in Chinese** |
| --- | --- |
| stool | 粪便（例如：次数是否正常；干稀是否正常） |
| verbal expression | 语言表达能力（例如：是否能清楚表达自己的想法等） |
| family (e.g.support；fights) | 家庭关系（例如：家庭支持；家庭矛盾等） |
| urinate | 小便（例如：次数是否正常；颜色是否正常） |
| grief | 是否感到悲伤 |
| constitution | 体质 |
| spirit of eye | 眼神（例如：目光是否有神；眼神转动是否灵活等） |
| sleep | 睡眠质量 |
| mobility | 行动能力；走动能力（如果平常使用拐杖或轮椅，那么按照使用时的情况来回答） |
| sound/hearing | 听力 |
| appetite | 食欲 |
| self-care | 自理能力，照顾自己的能力（例如：擦洗，穿衣，上厕所等） |
| discomfort | 是否感到不舒服，不适（例如：感觉想吐，喘不上气，瘙痒等；但不包括疼痛） |
| energy | 精力是否充沛 |
| pain | 是否感到疼痛 |
| complexion/color of face | 气色（例如：面色是否有光泽，口唇润泽等） |
| fatigue/exhausted | 是否感到疲劳 |
| joy/happy mood | 是否感到开心 |
| anxiety | 是否感到焦虑 |
| worry | 是否感到担忧 |
| confidence | 信心 |
| fear | 是否感到恐惧 |
| anger | 是否感到生气或愤怒 |
| memory | 记忆力 |
| concentration | 专注力 |
| loneliness | 是否感到孤独 |
| ability to complete work and study | 完成工作和学习的能力 |
| sex life | 性生活 |
| dwelling conditions | 居住条件 |
| economic conditions/status | 经济条件 |
| satisfaction | 是否对自己的生活感到满意 |
| climate adaptation and adjustment | 气候适应能力 (例如：对居住地气候的适应性；对季节气候变化的适应性等) |
| daily activities | 日常活动能力（例如：家务，休闲活动及娱乐，购物，旅行等） |
| heavy physical work | 重体力活（例如：搬运重物等） |
| body strength | 体力 |
| vision | 视力 |
| body weight | 体重 |
| family medical history | 家庭用药史 |
| physical exercise | 身体锻炼 |
| dependence on medication | 药物依赖（例如：日常生活是否需要依靠药物或医疗的帮助） |
| depression | 是否感到抑郁 |
| stress | 是否感到压力 |
| regularity in daily life | 日常生活的规律性 |
| morality | 品行；道德 |
| clear mind | 清晰的头脑或思维（可以清晰地思考） |
| positive attitude | 积极的态度（例如：积极的心态或生活态度） |
| sharp mind | 清晰的头脑或思维（可以快速反应） |
| peace | 内心平和 |
| breadth of mind | 心胸宽广（例如：包容，慷慨等） |
| social support | 社会支持 |
| feel unsafe | 是否感到不安全（例如：害怕跌倒，身体伤害或受到虐待） |
| ability to make decisions | 做决定的能力；我能自己做决定 |
| adaptability to social environment | 社会环境适应性（例如：应对不良刺激，适应嘈杂环境，与人打交道等） |
| frustrated | 是否感到沮丧 |
| social interactions | 社交（例如：与他人良好的关系或交流） |
| burdens to others | 是否感到自己是他人的负担 |
| had nothing to look forward to | 是否感觉没有什么事情可以期待 |
| have no control over day-to-day life | 是否能够掌控自己的日常生活（例如：是否可以选择自己做什么，或者别人对你做什么） |
| feel unable to cope with day-to-day life | 是否能够应对自己的日常生活 |
| feel good about yourself | 是否对自己感觉不错 |
| do the things you wanted to do | 是否能够做自己想做的事情 |
| feel accepted by others | 是否感到被他人接受（例如：是否能够做自己并且感到有归属感） |
| The items highlighted in blue are the dimensions of the EQ-5D  The items highlighted in yellow are the newly added items of EQ-HWB | |
