# Supplementary material for: The EQ-5D and EQ-HWB fit the perceptions of quality of life from a Chinese perspective: a concept mapping study
Source: Health Qual Life Outcomes. 2025 Mar 31;23:29. doi: 10.1186/s12955-025-02361-3 (PMC11959868; doi:10.1186/s12955-025-02361-3)
Supplement: Supplementary file 4 — Supplementary Material 4. [file 12955_2025_2361_MOESM4_ESM.docx]

# Appendix 4. Statements and Cluster Report in Netherlands

| **Cluster solution** | **Statements** | | **Bridging** |  | **Importance** |
| --- | --- | --- | --- | --- | --- |
| 1 Mental | | | 0.22 |  | 3.53 |
|  | 1 | feel accepted by others | 0.64 |  | 3.22 |
|  | 3 | feel good about yourself | 0.22 |  | 4.17 |
|  | 5 | have no control over day-to-day life | 0.45 |  | 3.83 |
|  | 6 | had nothing to look forward to | 0.08 |  | 3.67 |
|  | 7 | frustrated | 0 |  | 2.89 |
|  | 8 | feel unsafe | 0.22 |  | 3.22 |
|  | 9 | burdens to others | 0.37 |  | 3.11 |
|  | 14 | breadth of mind | 0.8 |  | 3.78 |
|  | 15 | peace | 0.19 |  | 3.89 |
|  | 16 | positive attitude | 0.41 |  | 4.33 |
|  | 21 | stress | 0.21 |  | 3.22 |
|  | 22 | depression | 0 |  | 3.89 |
|  | 35 | loneliness | 0.19 |  | 3.44 |
|  | 36 | satisfaction | 0.41 |  | 3.89 |
|  | 40 | confidence | 0.19 |  | 3.89 |
|  | 41 | anxiety | 0 |  | 3.5 |
|  | 42 | anger | 0 |  | 2.83 |
|  | 43 | worry | 0 |  | 2.94 |
|  | 44 | joy/happy mood | 0.21 |  | 4.28 |
|  | 45 | fear | 0 |  | 3.11 |
|  | 56 | grief | 0 |  | 3.06 |

| **Cluster solution** | **Statements** | | **Bridging** |  | **Importance** |
| --- | --- | --- | --- | --- | --- |
| 2 Society connections | | | 0.84 |  | 3.72 |
|  | 2 | do the things you wanted to do | 0.79 |  | 3.89 |
|  | 10 | adaptability to social environment | 0.68 |  | 3.28 |
|  | 11 | social support | 1 |  | 3.67 |
|  | 12 | social interactions | 0.84 |  | 3.83 |
|  | 13 | ability to make decisions | 0.78 |  | 3.94 |
|  | 17 | morality | 1 |  | 3.5 |
|  | 32 | economic conditions/status | 0.86 |  | 3.83 |
|  | 33 | ability to complete work and study | 0.73 |  | 4 |
|  | 34 | dwelling conditions | 0.91 |  | 3.61 |
|  | 55 | family e.g. support; fights | 0.8 |  | 3.67 |

| **Cluster solution** | **Statements** | | **Bridging** |  | **Importance** |
| --- | --- | --- | --- | --- | --- |
| 3 daily activity | | | 0.61 |  | 3.55 |
|  | 20 | regularity in daily life | 0.73 |  | 3.11 |
|  | 27 | heavy physical work | 0.4 |  | 2.11 |
|  | 28 | physical exercise and play | 0.48 |  | 3.78 |
|  | 30 | daily activities | 0.72 |  | 3.78 |
|  | 31 | climate adaptation and adjustment | 0.86 |  | 2.39 |
|  | 37 | sex life | 0.76 |  | 3.33 |
|  | 38 | concentration | 0.58 |  | 3.72 |
|  | 39 | memory | 0.42 |  | 3.78 |
|  | 51 | self-care | 0.65 |  | 4.67 |
|  | 52 | mobility | 0.38 |  | 4.78 |
|  | 58 | verbal expression | 0.77 |  | 3.61 |

| **Cluster solution** | **Statements** | | **Bridging** |  | **Importance** |
| --- | --- | --- | --- | --- | --- |
| 4 physical function | | | 0.38 |  | 3.50 |
|  | 4 | feel unable to cope with day-to-day life | 0.69 |  | 4.22 |
|  | 18 | sharp mind | 0.68 |  | 3.44 |
|  | 19 | clear mind | 0.63 |  | 3.94 |
|  | 23 | family medical history | 0.39 |  | 2.5 |
|  | 24 | body strength | 0.14 |  | 3.44 |
|  | 25 | dependence on medication | 0.6 |  | 3.06 |
|  | 26 | body weight | 0.11 |  | 3.22 |
|  | 29 | vision | 0.1 |  | 3.89 |
|  | 46 | fatigue/exhausted | 0.68 |  | 3.17 |
|  | 47 | complexion/color of face | 0.17 |  | 2.78 |
|  | 48 | discomfort | 0.7 |  | 3.94 |
|  | 49 | sound/hearing | 0.1 |  | 3.22 |
|  | 50 | pain | 0.67 |  | 4.06 |
|  | 53 | energy | 0.35 |  | 3.89 |
|  | 54 | appetite | 0.31 |  | 3.33 |
|  | 57 | spirit of eye | 0.48 |  | 2.89 |
|  | 59 | constitution | 0.12 |  | 2.94 |
|  | 60 | urinate | 0.15 |  | 3.83 |
|  | 61 | stool | 0.15 |  | 3.78 |
|  | 62 | sleep | 0.32 |  | 4.5 |
